# Interreader reproducibility of the Neck Imaging Reporting and Data system (NI-RADS) lexicon for the detection of residual/recurrent disease in treated head and neck squamous cell carcinoma (HNSCC)

**DOI:** 10.1186/s40644-020-00337-8

**Published:** 2020-08-18

**Authors:** Tougan Taha Abdelaziz, Ahmed Abdel Khalek Abdel Razk, Manar Maamoun Mohamed Ashour, Ahmed S. Abdelrahman

**Affiliations:** 1 Department of diagnostic radiology, faculty of medicine, Ain Shams University, Cairo, Egypt; 2 Department of diagnostic radiology, faculty of medicine, Mansoura University, Mansoura, Egypt

**Keywords:** NI-RADS lexicon, HNSCC, Cancer imaging, Inter-reader agreement

## Abstract

**Background:**

To evaluate the inter- and intrareader agreement and reproducibility of the NI-RADS scoring system and lexicon with contrast-enhanced computed tomography (CECT) and contrast-enhanced magnetic resonance imaging (CEMRI).

**Methods:**

This retrospective study included 97 CECT and CEMRI scans from 58 treated cases of head and neck squamous cell carcinoma (HNSCC) after the exclusion of head and neck cancers (HNCs) other than SCC and noncontrast and poor quality CT and MRI scans, with a total of 111 primary targets and 124 lymph node (LN) targets. Two experienced readers independently scored the likelihood of residual/recurrence for these targets based on the NI-RADS criteria and filled in report templates for NI-RADS lexicon diagnostic features. Inter- and intraobserver reproducibility was assessed with Cohen’s kappa, and the percent agreement was calculated.

**Results:**

Almost perfect interreader agreement was found for the final NI-RADS category of the primary lesions and LNs, with *K* = 0.808 and 0.806, respectively. Better agreement was found for CT than for MRI (*K* = 0.843 and 0.77, respectively, *P* value 0.001). There was almost perfect agreement for excluding tissue enhancement (*K* = 0.826, 95% CI = 0.658–0.993, *P* value 0.001), with a percent agreement of 96.4%, and substantial agreement for discrete nodular and diffuse mucosal enhancement (*K* = 0.826, 95% CI = 0.658–0.993, *P* value 0.001), with a percent agreement of 96.4%. There was fair agreement for focal mucosal nonmass and deep ill-defined enhancement. The intrareader agreement was almost perfect for most of the rated features (K ranging from 0.802 to 1), with the exception of enlarging discrete nodule/mass and focal mucosal nonmass-like enhancement, which had substantial intraobserver agreement (K ranging from 0.768 to 0.786).

**Conclusion:**

The individual features of NI-RADS show variable degrees of confidence; however, the overall NI-RADS category was not significantly affected.

## Background

Locoregional head and neck squamous cell carcinoma (HNSCC) recurrence is observed in 15–50% of patients and represents a central cause of disease morbidity and mortality [1]. Disfigurement of the normal anatomy and the soft tissue changes occurring mainly after treatment with surgery and radiotherapy complicate the interpretation of imaging findings [2]. The free-text reporting method varies by radiologists’ experience level and personal preference, a factor that may not answer modern clinical inquiries [3]. The imaging surveillance protocol for HNC varies among different institutes, and the radiologist’s impression is frequently uncertain and dissociated from the management recommendation. In addition, the interobserver agreement between radiologists regarding tumor recurrence and appropriate follow-up is unknown. In view of these obstacles, the American College of Radiology (ACR) released NI-RADS, a standardized report template associated with management recommendations [4]. This structured reporting approach can be simplified as a common language between radiologists and clinicians and a data-driven optimization of HNC imaging, with profitable results for patient management [5]. Furthermore, this reporting system serves in sharing data among different institutions, which may improve the research field in HNC [6].

There are limited but encouraging data concerning the baseline performance of NI-RADS for the evaluation of disease persistence/recurrence, interreader agreement, imaging modalities, and different time points of the surveillance protocol [7]. Although NI-RADS is currently adopted by several institutions, there is still limited evidence of whether the elaborated NI-RADS improves interreader agreement. In our opinion, structured reports should be a dynamic system, developed as the product of existing data and expert radiological and clinical consensus that will continue to be refined and updated as experience and validation data accumulate and in response to user feedback. To address this gap in knowledge, we designed this retrospective study involving CECT and CEMRI scans from treated patients with HNSCC.

The purpose of this study is to scrutinize the inter- and intrareader agreement of NI-RADS scoring for the likelihood of tumor residual/recurrence and to assess the interpretation reproducibility of NI-RADS lexicon imaging features using CECT and CEMRI in routine clinical practice.

## Methods

### Patients

Our institution ethics committee approved this single-center, retrospective study and waived the requirement for informed consent. Data were retrieved from medical records and PACS. The inclusion criteria were patients with HNSCC who finished their treatment and had been submitted to posttreatment imaging surveillance with either CECT or CEMRI according to our institution protocol, with no sex predilection. The exclusion criteria included HNCs other than SCC, scans other than CECT or CEMRI, scans not fulfilling the NI-RADS template requirements such as noncontrast-enhanced scans, and low-quality scans. An electronic search was performed in our hospital PACS system for the period from November 2017 to April 2019, yielding 500 CECT and 200 CEMRI examinations. A total of 97 scans from 58 patients met our inclusion and exclusion criteria, and these scans consisted of 45 CT and 52 MRI examinations. The following data were collected from the hospital medical records: demographic data, primary tumor site, initial stage (primary lesion, nodes and metastasis), and received treatment (chemotherapy, radiation, and surgery). Our institution posttreatment imaging surveillance protocol is a baseline scan 8 weeks after finishing treatment, followed by three-month follow-up intervals for 2 years.

### Image acquisition

i. CECT: Multidetector spiral CT, 128-row MDCT scanner (GE 128, Optima 660, USA), supine position, with arms down. FOV: 28 cm. One hundred milliliters of iodinated contrast agent was injected at a rate of 1–1.5 ml/sec, and scanning was initiated 80–100 s after the start of the contrast agent injection. Axial images ranged from the frontal sinuses down through the mediastinum. The native CT images were acquired with a slice thickness of 0.6–0.75 mm and reformatted for display with a slice thickness of 3 mm.
ii. CEMRI: 1.5 T MRI (Philips Ingenia scanner, Healthcare, Netherlands), using a dedicated head and neck coil. FOV AP 230, 4-mm slice thickness. Sequences: axial echo-planar DWI, precontrast axial T1WIs (TE/TR: 21/633 ms), coronal T1 (TE/TR: 14/555 ms), axial T2WIs (TE/TR: 10/7039 ms), sagittal T2WI (TE/TR: 100/3196.7 ms), and coronal T2 STIR (TR/TI = 3500/150, TE = 80 ms). Gadolinium (0.1 mmol/kg) was administered by using an injector with a flow rate of 2–3 ml/sec, which was followed by postcontrast T1WI with fat saturation in the axial (TR/TE: 611/21 ms), sagittal (TR/TE: 570/14 ms), and coronal (TR/TE: 570/14 ms) planes; total scan time: 18–25 min.

### Imaging analysis

The initial scan interpretation was performed by a 25-year experienced reader and yielded a total of 235 imaging targets: 111 primary targets and 124 LN targets. For precise interpretation, in cases of more than solitary, separate lesions, whether primary or nodal, lesions were defined with different numbers as primary target one and primary target two. Two head and neck radiologists with 15 and 11 years of experience served as reader 1 (R1) and reader 2 (R2). The readers had access to the patients’ initial scans performed before treatment and the patients’ demographic data, including age, sex, primary tumor site and the received treatment but were blinded to the patients’ names and the original imaging interpretation. The readers were directed to the targets through a series/image number, primary tumor site, and additional spatial identifying information if multiple lesions were present. DWI images were excluded from interpretation to solely assess the defined NI-RADS lexicon diagnostic features.

Per lesion agreement and per patient agreement were assessed by asking the readers to fill in a template for the enhancement and morphological features of each target using the NI-RADS lexicon CEMRI and CECT diagnostic features and to categorize the final NI-RADS score for the primary site and cervical LNs separately.

**Fig. 1:**
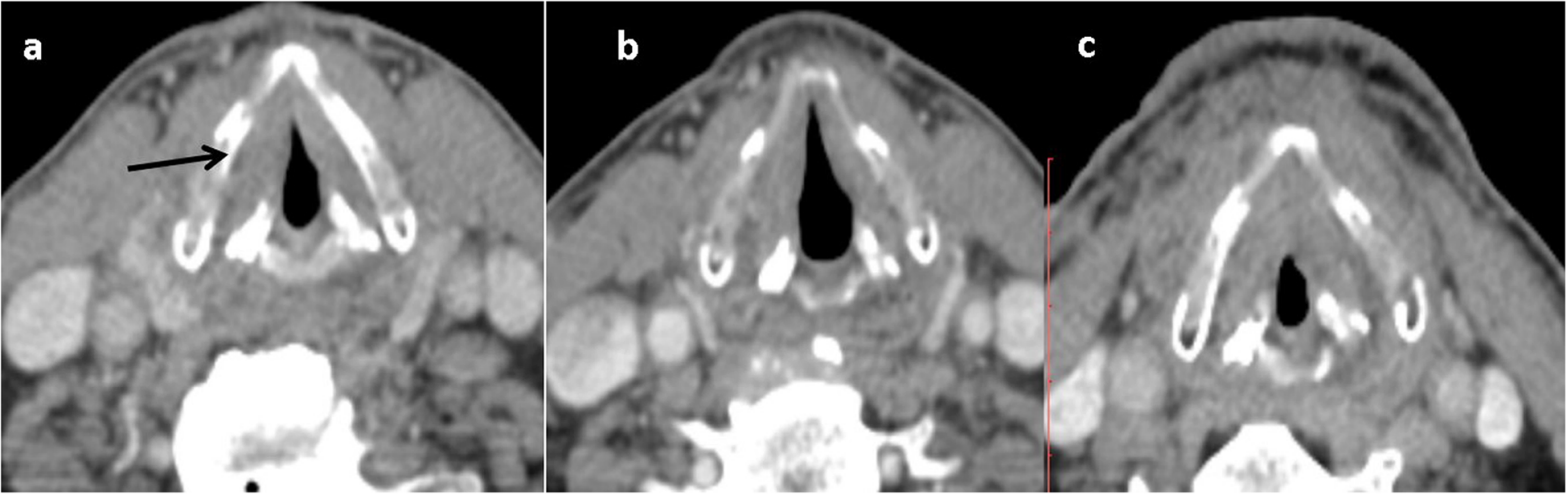
Primary NI-RADS 1, Neck NI-RADS 1 of cancer larynx. **a** Pretreatment axial CECT shows right glottic SCC. **b** First post treatment CT follow up after 3 months shows mild, smooth thickening of the Right vocal cord. **c** Second follow up after another 3 months shows progressive diffuse glottic thickening with minimal mucosal enhancement. No cervical LN. The patient was stable on his further follow up

**Fig. 2:**
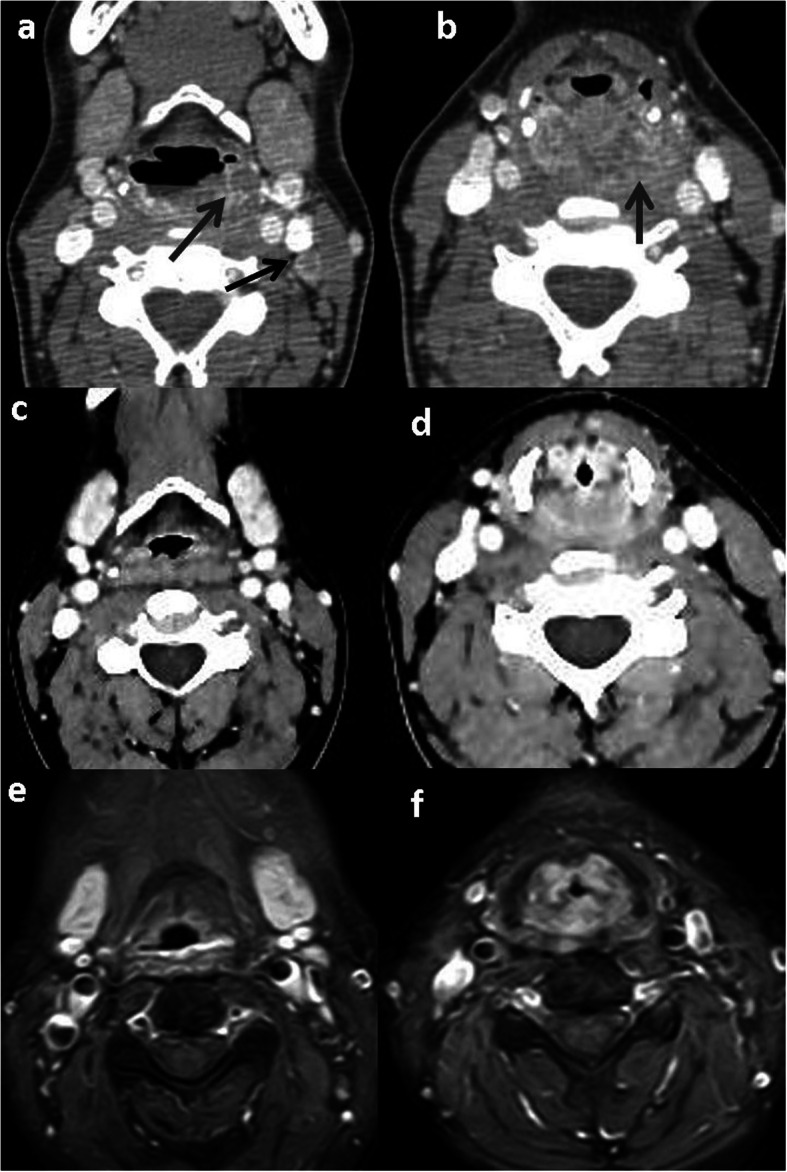
Primary NI-RADS 2, Neck NI-RADS 1 hypopharyngeal carcinoma: **a, b** Pre-treatment axial CECT shows hypopharyngeal SCC (grey arrows) Left malignant LN with cystic changes (black arrow). **c, d** first post treatment follow up CT shows diffuse hypopharyngeal thickening and enhancement. Resolution of the LN **e, f** Second follow up CEMRI axial images reveal stability of the Enhancement pattern

**Fig. 3:**
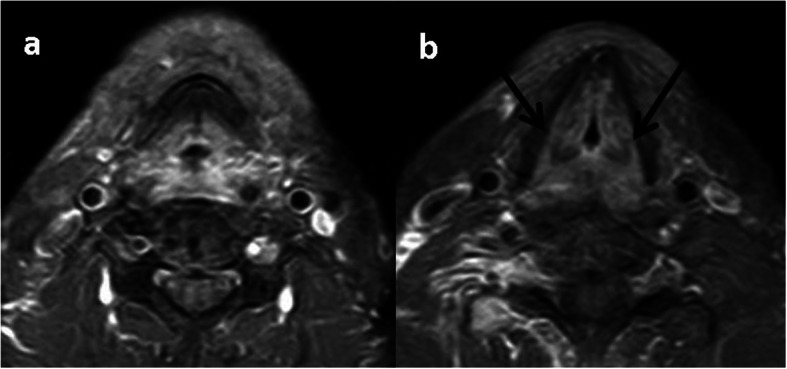
Primary NI-RADS 2, Neck NI-RADS 1 treated glottic SCC: axial CE T1WI (**a**) supra glottic (**b**) glottic larynx show diffuse non-mass, mucosal and submucosal enhancement with no focal lesions. No cervical LN. The patient was stable on further follow ups

**Fig. 4:**
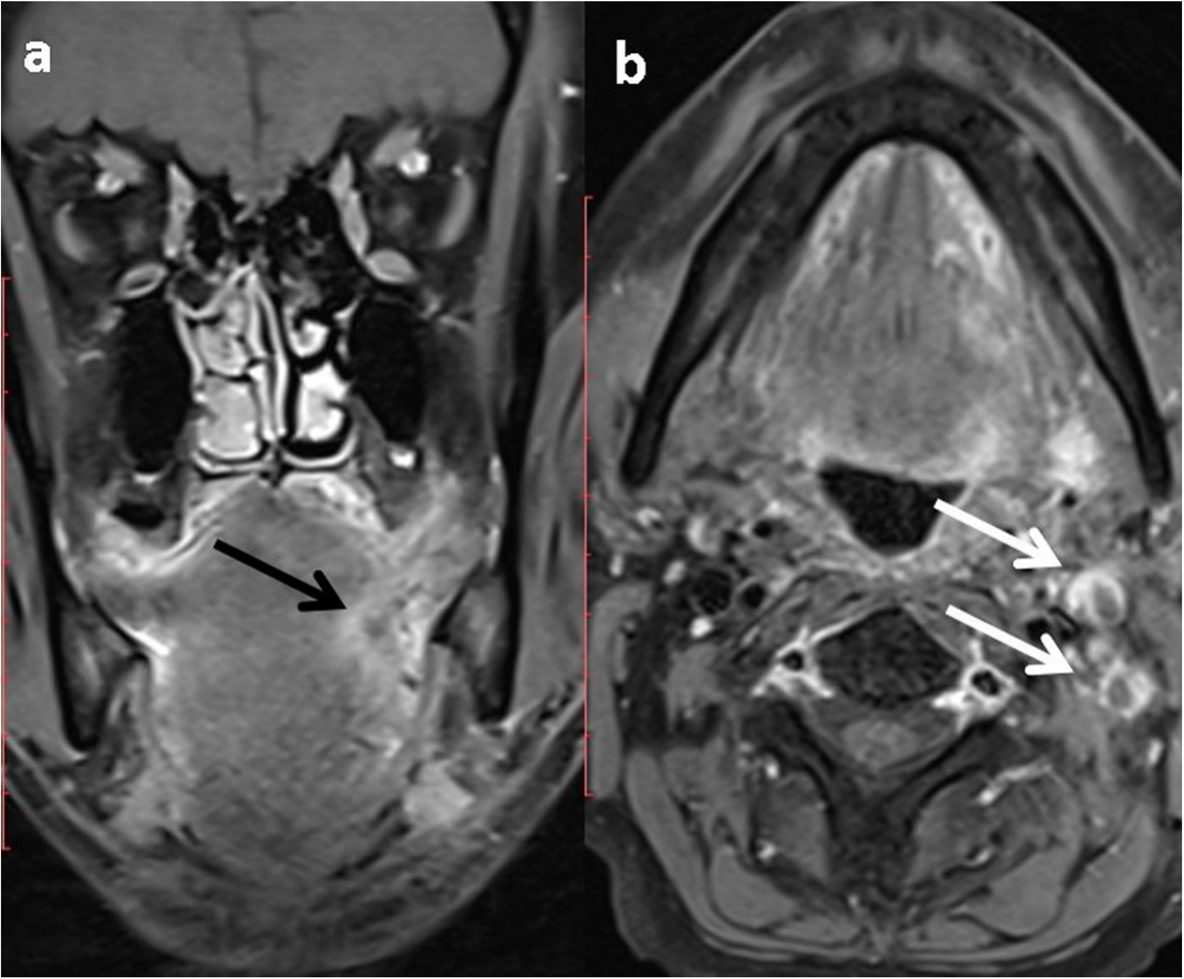
Primary NI-RADS 3, Neck NI-RADS 3 of Tongue SCC: post treatment CEMRI, coronal (**a**) and axial (**b**) show highly suspicious focal enhancing mass at left lateral tongue (black arrow). Left level II malignant looking LN with central cystic breaking down (white arrows)

**Fig. 5:**
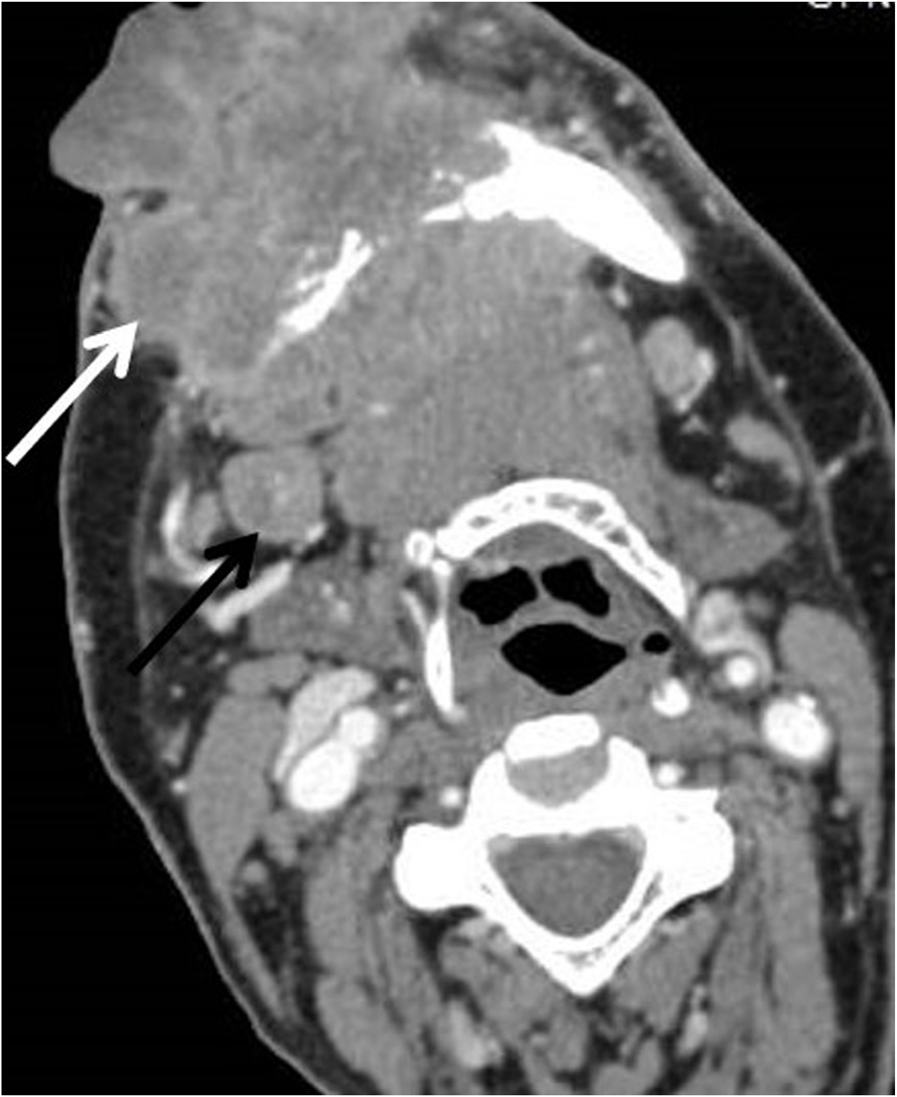
Primary NI-RADS 4, Neck NI-RADS 2 of lip SCC, post treatment CECT scan shows aggressive soft tissue mass invading the mandible with skin invasion (white arrows). Right level II LN with no definite suspicious morphology (Black arrow)

**Fig. 6:**
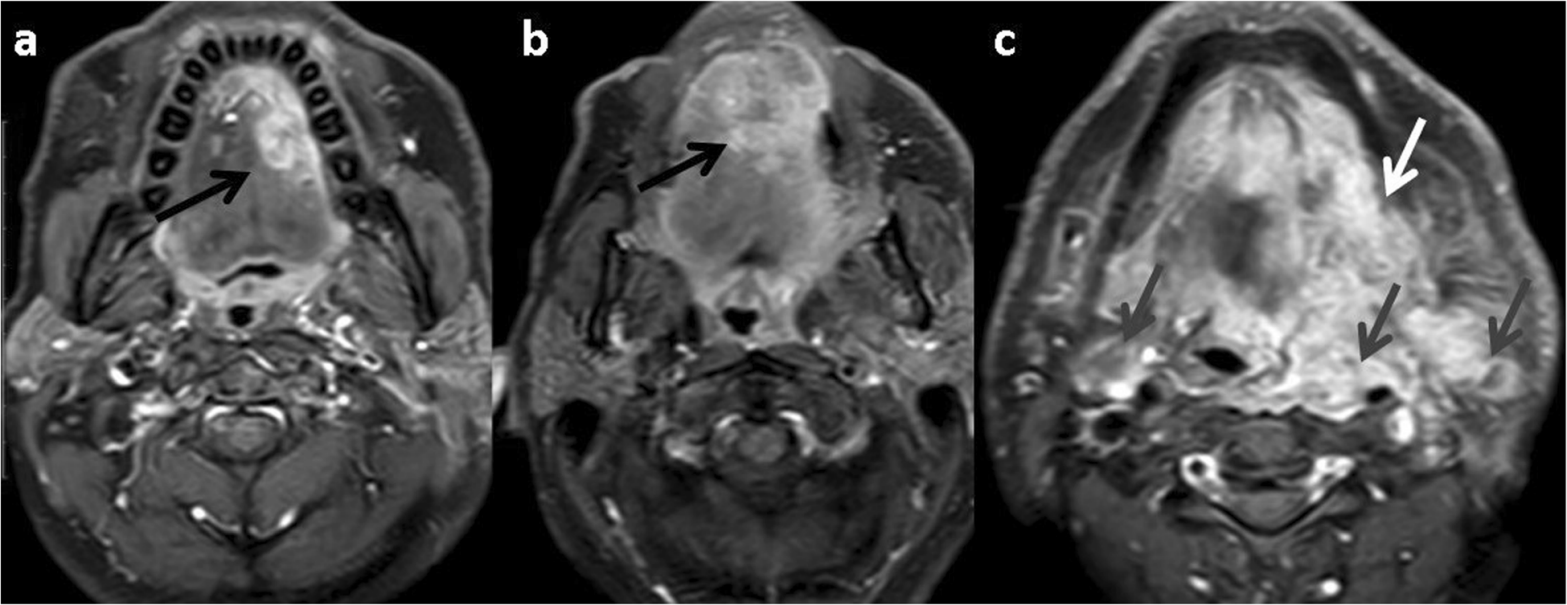
Primary NI-RADS 4, Neck NI-RADS 3 for tongue SCC: axial CEMRI (**a**) pretreatment, (**b**) first FU (**c**) second FU. Show the continuous progression of the left lateral tongue enhancing mass, crossing the midline in the first follow up (black arrows), and invading the floor of the mouth and pharyngeal wall in second follow up (white arrows). De novo bilateral malignant looking LN (grey arrows)

We used the ACR NI-RADS reporting template directions as follows: category 1 for no evidence of recurrence (Fig. 1); category 2 for low suspicion in the form of an ill-defined nonmass-like area or nondifferential enhancement (Figs. 2 and 3); category 3 for highly suspicious lesions with discrete, enlarging or new lesions with differential enhancement (Fig. 4); and category 4 for definite clinical or radiological progression (Figs. 5 and 6) [7]. In nodal disease, NI-RADS 1 showed no enlarging LNs and no new suspicious features (Fig. 2). A growing LN was categorized as NI-RADS 2 if there was no morphological abnormality (Fig. 5) and NI-RADS 3 (Figs. 4 and 6) if a morphological abnormality was present (necrosis or extranodal extension) [5]. NI-RADS scoring for the primary tumor site and cervical LNs were documented. The exact reporting system was repeated after 4 months by the two readers. The same target lesions were arranged in a newly randomized way to avoid bias from the previous results.

### Statistical analysis

The data were input into the computer and analyzed using Statistical Package for Social Science (IBM Corp, released 2013; IBM SPSS statistics for Windows, V. 22.0; Armonk, NY, USA) and GraphPad QuickCalcs (https://www.grapghpad.com/quickcalcs/kappa1/). Qualitative data are described using numbers and percentages.

Binary variables were used for the morphological and enhancement categorical data, and an ordinal variable was used for the NI-RADS categorical data score. Cross-tabulation for the categorical variables was performed with an estimation of the frequency of each categorical variable. The percent of agreement was estimated as a percent of concordance. Cohen’s kappa coefficient (κ) was calculated to test the interreader and intrareader agreement and the 95% confidence intervals were calculated. The kappa test was statistically significant when the *p* value was less than 0.05. Kappa agreement was interpreted as follows: 0.01–0.20: slight agreement; 0.21–0.40: fair agreement; 0.41–0.60: moderate agreement; 0.61–0.80: substantial agreement; and 0.81–0.99: almost perfect agreement).

## Results

A total of 97 scans from fifty-eight treated HNSCC patients comprising 45 CT scans and 52 MRI scans were examined. These scans were performed at different time points within the initial 2 years of posttreatment follow-up. The tumor subsets of the included patients were 39 oral cavity carcinomas, 38 laryngeal carcinomas, 10 nasopharyngeal carcinomas, 5 sinonasal carcinomas, 3 hypopharyngeal carcinomas, 1 skin neoplastic lesion, and 1 sublingual salivary gland carcinoma. Regarding patient demographics, 38 (65.5%) patients were female and 20 (34.5%) patients were male, with a mean age of 52.66 ± 13.75 years (range 18 to 89 years).

In this study, we analyzed the per lesion agreement for NI-RADS lexicon features for the included primary and nodal targets, and we found almost perfect agreement between the two observers in excluding any enhancement of the primary neck lesion, with *K* = 0.826, (CI 95%, 0.658–0.993) (*P* value < 0.001) and a percent agreement of 96.4%, yet a substantial agreement was found in detection of either discrete nodular enhancement or diffuse linear mucosal enhancement, with *K* = 0.730 and 0.706, respectively (*P* value < 0.001). Regarding the detection of either focal mucosal nonmass-like enhancement or deep ill-defined enhancement, our study showed fair interobserver agreement (*K* = 0.309 and 0.247, respectively) (Table 1).

**Table 1:** Inter-observer agreement of the morphological findings for the 111 primary tumor sites

Morphological and enhancement pattern	Reader 1	Reader 2	Matched cases between reader 1 &2	K	95% CI	***P*** value	Percent agreement
Discrete nodular enhancement.	53	56	47	0.730	0.603–0.857	< 0.001*	86.5%
Definite progressive mass.	25	13	12	0.564	0.369–0.760	< 0.001*	87.39%
New or enlarging discrete nodule/ mass	34	40	19	0.273	0.087–0.458	0.004*	67.57%
Deep ill-defined enhancement.	63	24	21	0.247	0.116–0.378	0.001*	59.46%
Deep, ill-defined soft tissue mass.	59	19	14	0.135	0.004–0.266	0.049*	54.95%
Focal mucosal non-mass like enhancement.	16	6	4	0.309	0.05–0.568	< 0.001*	87.39%
No enhancement.	14	12	11	0.826	0.658–0.993	< 0.001*	96.4%
Diffuse linear mucosal enhancement.	31	30	24	0.706	0.56–0.856	< 0.001*	88.3%
Non-mass like tissue distortion.	67	49	41	0.402	0.242–0.562	< 0.001*	69.37%
Low density mucoid mucosal edema.	22	22	14	0.546	0.350–0.743	< 0.001*	85.6%

For the morphological features, we found moderate interobserver agreement for definite progressive mass lesions, low-density mucosal edema and nonmass-like tissue distortion, with *K* = 0.564, 0.546 and 0.402, respectively (*P* value < 0.001), yet much less agreement was found between the two readers in the detection of deep, ill-defined nondiscrete soft tissue, with *K* = 0.135 (*P* value< 0.049) (Table 1).

Almost perfect *per lesion* interobserver agreement was noted for the CT and MRI of the primary and neck LN lesions, with *K* = 0.819 and 0.848, respectively (CI 95%, 0.731–0.908 and 0.761–0.935, respectively, *P* value < 0.001) (Tables 2 and 3).

**Table 2:** Inter-observer agreement of the CT & MRI & cross section examination per lesion (P lesion) and per patient (P patient) for the primary tumor site

Groups	***K***	95% CI	***P*** value	Percent agreement
**P lesion: CT (No = 51)**	0.862	0.748–0.977	< 0.001*	90.2%
**P lesion: MRI (No = 60)**	0.778	0.645–0.912	< 0.001*	85.0%
**P lesion: Cross section (No = 111)**	0.819	0.731–0.908	< 0.001*	87.39%
**P lesion for oral cavity: Cross section (No = 39)**	0.80	0.647–0.953	< 0.001*	82.1%
**P lesion for larynx: Cross section (No = 38)**	0.92	0.817–1.0	< 0.001*	94.7%
**P patient: CT (No = 45)**	0.843	0.713–0.973	< 0.001*	88.9%
**P patient: MRI (No = 52)**	0.77	0.627–0.917	< 0.001*	84.62%
**P patient: Cross section (No = 97)**	0.808	0.710–0.904	< 0.001*	86.6%

**Table 3:** Inter-observer agreement of the CT & MRI & cross section examination per lesion (P lesion) and per patient scan (P patient) for LN

Groups	***K***	95% CI	***P*** value	Percent agreement
**LN P lesion: CT (No = 55)**	0.843	0.695–0.991	< 0.001	92.73%
**LN P lesion: MRI (No = 69)**	0.849	0.734–0.965	< 0.001	91.3%
**LN P lesion: Cross section (No = 124)**	0.848	0.761–0.935	< 0.001	91.94%
**LN P patient: CT (No = 45)**	0.867	0.687–0.99	< 0.001	95.6%
**LN P patient: MRI (No = 52)**	0.816	0.642–0.989	< 0.001	92.3%
**LN P patient: Cross section (No = 97)**	0.806	0.668–0.945	< 0.001	92.8%

The *per scan* interobserver agreement of cross-sectional imaging including both CT and MRI examinations for the primary and lymph node lesions was almost perfect, with *K* = 0.808 and 0.806, respectively (Tables 2 and 3).

Slightly less per lesion and per scan interobserver agreement was seen in the MRI examination of the primary neck lesions, with *K* = 0.778 and 0.77, respectively (*P* value < 0.001), yet a higher agreement was seen in the CT examination, with *K* = 0.862 and 0.843, respectively (*P* value < 0.001).

A near similar agreement was observed in the CT and MRI studies for the description of LNs regarding the per lesion and per scan analyses (*K* = 0.843 and 0.867 for CT; *K* = 0.849 and 0.816 for MRI; *P* value < 0.001 (Tables 2 and 3).

Almost perfect interobserver agreement was found between the two readers for the discrimination of the oral cavity and laryngeal carcinoma, with *K* = 0.8 and 0.92, respectively (CI 95%, 0.647–0.953 and 0.817–1.0, respectively, *P* value < 0.001), yet the agreement of other areas could not be calculated due to a reduced number of scans or missing primary NI-RADS categories (Table 2).

Substantial intraobserver agreement was found for both readers for the detection of new or enlarging discrete nodule/mass morphology, with *K* = 0.776 and 0.768 (CI 95%, 0.650–0.911 and 0.644–0.891, *P* value < 0.001) and a percent agreement of 90.1 and 89.2% for R1 and R2, respectively (Tables 4 and 5).

**Table 4:** Intra-observer agreement for the morphological findings for the 111 primary lesions

Morphological and enhancement pattern	Reader	First reading	Second reading	Matched cases between 1st & 2nd reading	K	95% CI	***P*** value	Percent agreement
Discrete nodular enhancement.	1	53	53	48	0.819	0.713–0.925	< 0.001*	95.4%
	2	56	62	55	0.856	0.760–0.952	< 0.001*	92.7%
Definite progressive mass.	1	25	26	22	0.822	0.694–0.949	< 0.001*	93.7%
	2	13	15	12	0.837	0.682–0.992	< 0.001*	92.8%
New or enlarging discrete nodule mass	1	34	39	31	0.776	0.650–0.911	< 0.001*	90.1%
	2	40	42	35	0.768	0.644–0.891	< 0.001*	89.2%
Deep ill-defined enhancement.	1	63	56	54	0.802	0.795–0.983	< 0.001*	90%
	2	24	27	22	0.822	0.695–0.949	< 0.001*	93.7%
Deep, ill-defined soft tissue mass.	1	59	59	54	0.819	0.711–0.927	< 0.001*	90.9%
	2	19	23	18	0.824	0.688–0.959	< 0.001*	94.6%
Focal mucosal non-mass like enhancement.	1	16	19	15	0.831	0.687–0.974	< 0.001*	87.39%
	2	6	9	6	0.786	0.552–1.0	< 0.001*	97.3%
No enhancement.	1	14	14	14	1	1–1	< 0.001*	100%
	2	12	11	11	0.952	0.857–1.0	< 0.001*	99.1%
Diffuse linear mucosal enhancement.	1	31	32	29	0.889	0.794–0.983	< 0.001*	95.5%
	2	30	31	28	0.887	0.790–0.983	< 0.001*	95.5%
Non-mass like tissue distortion.	1	67	66	64	0.906	0.825–0.986	< 0.001*	95.5%
	2	49	49	45	0.854	0.756–0.952	< 0.001*	92.8%
Low density mucoid mucosal edema.	1	22	20	19	0.883	0.994–0.771	< 0.001*	96.4%
	2	22	22	19	0.830	0.698–0.961	< 0.001*	94.6%

**Table 5:** Intra-observer agreement of the CT & MRI & cross section examination per patient (P patient) for the primary tumor site and LN

Groups	Reader	***K***	95% CI	***P*** value	Percent agreement
**P patient: CT (No = 45)**	1	0.813	0.676–0.950	< 0.001*	86.6%
	2	0.783	0.634–0.931	< 0.001*	84.5%
**P patient: MRI (No = 52)**	1	0.915	0.820–1.0	< 0.001*	94.2%
	2	0.884	0.774–0.993	< 0.001*	92.2%
**P patient: Cross section (No = 97)**	1	0.867	0.784–0.949	< 0.001*	90.7%
	2	0.838	0.747–0.928	< 0.001*	88.6%
**P lesion for oral cavity: Cross section (No = 39)**	1	0.879	0.747–1.0	< 0.001*	92.3%
	2	0.836	0.685–0.986	< 0.001*	89.8%
**P lesion for larynx: Cross section (No = 38)**	1	0.883	0.757–1.0	< 0.001*	92.2%
	2	0.848	0.706–0.989	< 0.001*	89.5%
**LN P patient Scan: CT (No = 45)**	1	1	1–1	< 0.001*	100%
	2	0.873	0.710–1.0	< 0.001*	95.6%
**LN P patient Scan: MRI (No = 52)**	1	0.755	0.567–0.943	< 0.001*	83.9%
	2	0.834	0.681–0.986	< 0.001*	92.3%
**LN P patient Scan: Cross section (No = 97)**	1	0.860	0.744–0.975	< 0.001*	94.9%
	2	0.851	0.739–0.962	< 0.001*	93.9%

R2 (less experienced reader) showed a substantial intraobserver agreement for the discrimination of the primary neck lesion by CT examination and for the detection of the enhancement pattern of focal mucosal nonmass like enhancement, with *K* = 0.783 and 0.786 and a percent agreement of 84.5 and 97.3%, respectively (*P* value < 0.001), yet R1 showed substantial intraobserver agreement for the distinction of LN lesions in the MRI examination, with *K* = 0.755 (*P* value < 0.001) and a percent agreement of 83.9%.

The two readers showed an almost perfect intraobserver agreement regarding the other rated features, with K values ranging from 0.802 to 1 and percent agreement values ranging from 86.6 to 100%.

## Discussion

The advances in treatment options and salvage procedures make free-text radiological reports no longer suitable for modern clinical inquiries. Structured reports have become a must to unify the radiological language and fulfill the key data elements, the quantified parameters, and the clinicians’ inquires [8]. A structured reporting system allows the assimilation of relevant information and recommendations contingent upon the currently available literature, and the incorporation of these data with clinical and radiological findings not only allows a precise diagnosis but also affects further clinical management decisions [9].

The identification of recurrent superficial growing tumors could be problematic in CT and MRI; however, these tumors could be easily detected by clinical examination where imaging is required to evaluate the depth of tumor invasion. CT and MRI are more valuable in the detection of tumor recurrence in head and neck regions that are not completely convenient by clinical examination. An enhancing and infiltrating soft tissue mass is the established imaging finding for positive head and neck tumor residual/recurrence [10]; however, in clinical practice, not all patients present with these obvious diagnostic features, and different patterns of morphological tissue abnormalities and enhancement appear, which should be categorized as recurrent disease or posttreatment changes. The combination of morphology and enhancement patterns in addition to the rate of growth is integral to imaging algorithms. NI-RADS promotes standardization and therefore reproducibility across radiologists.

To our knowledge, few studies have evaluated the recently introduced NI-RADS [6, 7]; however, these few different studies have assessed the accuracy and overall performance of NI-RADS without introducing the NI-RADS lexicon. Gaps in knowledge remain regarding the interreader agreement of individual features of the NI-RADS lexicon and their integration into a structured algorithm. In this study, we categorized different tissue morphology and enhancement patterns according to the NI-RADS lexicon and evaluated the interreader agreement for the different features that affect the agreement for the lesion and therefore the overall NI-RADS category of the patient.

We assessed the NI-RADS interreader reliability for the CECT and CEMRI templates. NI-RADS was initially developed for surveillance with CECT and then adapted to PET/CT and was finally modified for CEMRI scans. In our institution, the choice of imaging modality depends on the tumor site to be evaluated, the proposed clinical inquiries, and the patients’ clinical status in an individualized manner.

Considering the enhancement patterns, our study revealed significant interreader agreement for excluding tissue enhancement, the detection of discrete nodules, and linear mucosal enhancement patterns. However, the readers were skeptical about focal mucosal nonmass and deep ill-defined enhancement patterns. In a previous study, Nooij et al. reported that the most controversy for the primary site occurs when both the recurrent tumor and the treatment-induced inflammation show a high T2 signal and postcontrast enhancement [11].

In the current study, the morphological features of NI-RADS showed a lower degree of confidence for nonmass like tissue distortion and deep ill-defined soft tissue masses; this is likely due to the overlap between recurrent tumor and posttreatment changes or benign complications. Recent studies have described the potential role of functional imaging to see the overall picture of posttreatment changes. M. Lell et al. reported that using conventional features such as morphology and enhancement makes it difficult to differentiate a residual or recurrent tumor from posttreatment changes, especially in the early posttreatment interval, resulting in a 46% false positive diagnosis. They found that with longer follow-up intervals, the posttreatment changes decreased, and the diagnosis was more definite [10]. Another study by Van der Hoorn et al. described the limited role of conventional MRI in posttreatment evaluation, which had a sensitivity of 84% and a specificity of 82% for local treatment response evaluation [12]. For these reasons, NI-RADS combines semiquantitative readouts based on observation recognition and quantitative measures. This combination led to a narrow change regarding the final NI-RADS category despite the varying levels of consensus for the individual features. In this study, there was almost perfect interreader agreement for the NI-RADS category for the primary tumor site, which was in agreement with the findings of a study performed by Krieger et al., which showed very good interobserver agreement of 0.821 (95% CI, 0.657–0.986) with *P* < 0.001 [7]. The other quantitative imaging biomarkers, such as diffusion, perfusion and spectroscopy, were not included in the NI-RADS algorithms.

The malignant criteria for nodal disease discussed in previous studies included increased axial diameter more than 10 mm, morphological changes such as attaining a rounded shape, regional grouping, presence of necrosis, grouping, and extracapsular spread of the tumor [13]. Lymph node assessment is impeded by reactionary changes, as reactive LNs may show borderline features; moreover, normally sized nodes can still contain malignant infiltration. Using size criteria for the evaluation of LN infiltration is confusing and unreliable because there are multiple size criteria reported in the literature [14]. In a study performed by Aiken et al., they found that cross-sectional imaging has high specificity for extracapsular spread (88%) and low sensitivity (68%), and the presence of necrosis was the best predictor for extracapsular tumor spread (*p* = 0.001), which had a significant *P* value compared with irregular border (*p* = 0.055) and gross invasion (*p* = 0.068) [15]. NI-RADS combines the size, morphology and functional features of cervical LNs in the follow-up algorithm. The current study revealed perfect per scan interobserver agreement for cross-sectional imaging, including both CT and MRI examination for both primary and lymph node lesions.

MRI reverses biochemical tissue characteristics and could improve tissue resolution compared to CT. Detrimentally, the scan times are relatively long during which the patient must remain still. Modern multislice CT machines allow short scan times without motion artifacts, a precious advantage in HNC patients who may have difficulty breathing, swallowing secretions, and lying flat [16]. In this study, better interobserver agreement was found in CT for the per lesion and per scan evaluations of the primary site; however, it did not significantly exceed the MRI agreement. Furthermore, a near similar agreement between the two modalities was found for LN evaluation.

There is a large geographical difference regarding the incidence of the primary site of head and neck cancers, which varies according to the prevalence of risk factors, ethnic and genetic differences among populations, and environmental factors such as diet and lifestyle [17]. In the current study, the oral cavity and larynx were the most common primary sites with an almost perfect interobserver agreement for the different NI-RADS categories.

Finally, there was satisfactory agreement between the head and neck radiologists for the final NI-RADS categories, and the variability in the morphological features could be overcome by adding functional imaging such as PET/CT, which is already included in the NI-RADS algorithms. The other quantitative imaging biomarkers not included in NI-RADS show the potential to provide decision support tools in the management pathway of head and neck cancer.

### Limitations

Our work encompassed all types of HNSCC collectively instead of investigating each subsite separately. Another limitation is the combination of subsequent CT and MRI scans in our study when ideally each modality should have been scrutinized separately. This study lacks functional imaging and an evaluation of the biological features of the treated tissue, which is expected to decrease the debate and increase the radiologist confidence about treated HNC imaging. The last limitation is the limited number of patients, as investigating a larger cohort of patients in a multicenter study is recommended.

## Conclusion

The interreader agreement was almost perfect for the final NI-RADS categorization of primary lesions and nodal disease. Different degrees of confidence were encountered regarding the morphology and enhancement features, which can be attributed to the overlap between posttreatment changes or benign complications and recurrent disease; however, this did not affect the overall NI-RADS category and the final recommendation for the next step in follow-up or clinical management.

## Data Availability

Not applicable.

## Abbreviations

NI-RADS: Neck imaging reporting and data system
HNSCC: Head and neck squamous cell carcinoma
HNC: Head and neck cancer
ACR: American college of radiology
LN: Lymph node
CEMRI: Contrast enhanced magnetic resonance imaging
CECT: Contrast enhanced computed tomography
